# CpGV-M Replication in Type I Resistant Insects: Helper Virus and Order of Ingestion Are Important

**DOI:** 10.3390/v13091695

**Published:** 2021-08-26

**Authors:** Aurélie Hinsberger, Christine Blachère-Lopez, Caroline Knox, Sean Moore, Tamryn Marsberg, Miguel Lopez-Ferber

**Affiliations:** 1 Hydrosciences Montpellier, Univ. Montpellier, IMT Mines Ales, IRD, CNRS, 6 Avenue de Clavières, 30319 Alès, France; aurelie.hinsberger@mines-ales.fr (A.H.); christine.blachere-lopez@mines-ales.fr (C.B.-L.); 2 INRAE, SPE, 400 Route des Chappes BP 167, CEDEX, 06903 Sophia-Antipolis, France; 3 Department of Biochemistry and Microbiology, Rhodes University, Makhanda 6139, South Africa; caroline.knox@ru.ac.za; 4 Centre for Biological Control, Department of Entomology and Zoology, Rhodes University, Makhanda 6139, South Africa; seanmoore@cri.co.za; 5 Citrus Research International, 63 Heugh Road, Walmer, Port Elizabeth 6070, South Africa; tammy@cri.co.za

**Keywords:** *Cydia pomonella*, resistance type I, resistance mechanisms, CpGV, CrpeNPV, baculoviruses, multiple infections

## Abstract

The genetic diversity of baculoviruses provides a sustainable agronomic solution when resistance to biopesticides seems to be on the rise. This genetic diversity promotes insect infection by several genotypes (i.e., multiple infections) that are more likely to kill the host. However, the mechanism and regulation of these virus interactions are still poorly understood. In this article, we focused on baculoviruses infecting the codling moth, *Cydia pomonella*: two Cydia pomonella granulovirus genotypes, CpGV-M and CpGV-R5, and *Cryptophlebia peltastica* nucleopolyhedrovirus (CrpeNPV). The influence of the order of ingestion of the virus genotypes, the existence of an ingestion delay between the genotypes and the specificity of each genotype involved in the success of multiple infection were studied in the case of *Cydia pomonella* resistance. To obtain a multiple infection in resistant insects, the order of ingestion is a key factor, but the delay for ingestion of the second virus is not. CrpeNPV cannot substitute CpGV-R5 to allow replication of CpGV-M.

## 1. Introduction

The use of pathogens is a well-known approach for biological control of insect pests. Among them, members of the baculovirus family are widely used as crop protection products [1,2].

The most common way of infection for baculovirus-based biocontrol products is the oral ingestion of the virus by host larvae. Baculoviruses produce occlusion bodies (OBs) that facilitate the survival and transmission of the virus from one host to another. Two types of OB can be found in baculovirus, polyhedra and granules. Polyhedra are found in the genera *Alphabaculovirus*, *Gammabaculovirus* and *Deltabaculovirus*, previously classified as nucleopolyhedroviruses (NPVs), while granules are found only in the *Betabaculovirus* that are also called granuloviruses (GVs). Polyhedra contain tens of virions while granules usually contain one (or sometimes more) virion [3,4]. When an OB is ingested, the basic pH conditions of the insect midgut induce its solubilisation and liberation of the virions they contain, called occlusion derived viruses (ODVs). In some alphabaculoviruses, called multiple nucleopolyhedroviruses (MNPV), ODVs can be polyploid, each ODV containing a variable number of nucleocapsids [5]. Each nucleocapsid contains a genome. Baculovirus genomes are dsDNA, circular, and around 100 kbp in size. Complete or defective genomes can be present in a nucleocapsid.

Baculoviruses show a high level of genetic diversity. In a given virus population, diverse genotypes can be found. In addition, geographically distant virus populations can differ not only in the genotypes present, but also in their respective frequencies [6,7].

In various MNPV, like *Spodoptera frugiperda* MNPV (SfMNPV), or *Autographa californica* MNPV (AcMNPV), it has been demonstrated that various genotypes can be occluded in a single OB [8]. This observation is likely true for all NPVs. Even more, non-identical viral genome copies can be contained in a single polyploid ODV [9]. In such conditions, ingesting a single OB can result in the infection of a larva by various genotypes. In GVs, as OBs usually contain a single virion, a multiple infection requires the larva ingesting several OBs containing different viral genotypes. It has been proposed that this multiple infection strategy is at the origin of the higher genetic diversity observed in the alphabaculoviruses compared to betabaculoviruses [8].

The various genotypes of a virus population differ in their biological characteristics, particularly the ability to overcome host defense mechanisms [10,11]. Genetic diversity of lepidopteran baculoviruses can be an asset for agronomic use, possibly even limiting the emergence of resistance. This genetic diversity can be exploited by alternating the genotypes used in the field, either on a temporal or on a spatial scale. Another approach is the use of inocula containing multiple genotypes. A given insect larva challenged with a mixture of genotypes can be infected by only one or by many. The success of infection will be a function of (i) the probability of a virus genotype being ingested, (ii) the insect’s own genetic makeup (the ability to allow or block the replication of a given virus genotype) and (iii) the interactions between genotypes inside the host. In some virus/host systems, only one genotype was found in each insect larva [12] while in others, multiple genotypes have been isolated from a single larva [13,14]. We call it multiple infection when there is viral diversity within the same host, and we reserve use of the term co-infection for infection of a single cell by different viruses [15]. For an OB to contain multiple genotypes, co-infection must occur.

In SfMNPV, the genetic diversity of isolates is maintained and finely regulated during the different generations to guarantee the best efficiency. In multiple SfMNPV infections, each genotype appears to contribute to preserve the survival of the whole population [16,17,18]. In the potato tuber moth, *Phthorimaea operculella* (Zeller) (Lepidoptera: Gelechiidae) it has been shown that some granulovirus isolates, which are mixtures of genotypes, have a higher pathogenicity than isolates composed of pure genotypes. It has also been shown that such association of different *Phthorimaea operculella* granulovirus (PhopGV) genotypes does not result in a “hybrid” genotype but that genotypes are maintained in stable frequencies over time [19,20].

CpGV is a granulovirus (of the genus *Betabaculovirus*) used as a bioinsecticide worldwide in the biological control of the codling moth, *Cydia pomonella* (Linnaeus) (Lepidoptera: Tortricidae), a pest of apples and pears. The first isolate of CpGV was found in Mexico. It is composed of a single genotype called CpGV-M. This isolate was used to develop commercial bioinsecticides extensively used in Europe since their approval in the nineties. However, after years of using CpGV-M, codling moth populations have developed resistance [21,22]. Following this observation, the analysis of the natural diversity in virus populations allowed the isolation of CpGV genotypes overcoming this resistance, among them, CpGV-R5 [23]. Today, the known diversity of CpGV is grouped in seven phylogenetic lines (A to F) [24], CpGV-M and CpGV-R5 belonging to the A and E group, respectively [25].

Five types of resistance in *C. pomonella* have been described [26]. They differ by the mode of transmission (linked to the Z chromosome or autosomal), and by the CpGV genotypes they can restrict. Type I resistance is specific to CpGV-M (A group) and sex-linked (located on the Z chromosome). Type II resistance is dominant, autosomal and reduces susceptibility to CpGV-C, -D, and -E genotype groups. Type III resistance shows a mixed type of transmission, autosomal and Z-linked. All but CpGV-B genotype groups are restricted. Resistance type IV blocks replication of A and B genotypes, but not of E genotypes. Finally, resistance type V blocks replication of all genotypes tested (A, B, E). In type I resistant insects (specific to the CpGV-M isolate), CpGV-M is unable to replicate in intestinal cells, but still crosses the peritrophic membrane and enters into the cells [27]. In laboratory conditions, when type I resistant larvae (laboratory colony R_GV_) were allowed to feed on mixtures of CpGV-M and CpGV-R5, successful replication of CpGV-M was detected. Surprisingly, these mixed virus populations appear to be more effective in controlling R_GV_ resistant individuals than CpGV-R5 alone [28]. Similar results were reported with other type I resistant laboratory colonies [25]. In our work, we aim to shed light on the process allowing such CpGV-M replication. It seemed important to know at which step of infection CpGV-R5 helps CpGV-M. After OB ingestion, the virus must pass the host intestinal barrier, before being able to colonize the fat body, its major replication tissue. Because CpGV is a GV, this implies that CpGV-R5 and CpGV-M are ingested separately, in different OBs. The first parameter analyzed was to determine if there was an order of ingestion for a double infection. The second was to determine if there was a delay between the ingestion of one then the other, during which the larva remains susceptible to the second virus.

Once the order and the delay of ingestion was characterized, it was necessary to determine (i) if the help needed for CpGV-M replication in a non-permissive insect is generic, for example by weakening the host, making it permissive to a second infection, regardless of the first pathogen, or (ii) the help provided to CpGV-M is specific to CpGV-R5. To that end, we tested the ability of another baculovirus to act as helper for CpGV-M. The *Cryptophlebia peltastica* nucleopolyhedrovirus (CrpeNPV) [29] is able to effectively replicate in codling moth [30], therefore can be used in the study to determine the helper effect in mixed infections.

## 2. Materials and Methods

### 2.1. Insects

CpNPP is the reference insect colony susceptible to CpGV-M and was provided by Natural Plant Protection (UPL-LTD, Noguères, France). It originates from Northern France, and is used for the industrial production of Carpovirusine^®^. CpNPP is a colony susceptible to CpGV-M and CpGV-R5 [28]. The susceptibility of CpNPP to CrpeNPV has been tested in the present work.

The R_GV_ insect colony is resistant to CpGV-M. This colony originates from a natural insect population collected in the field at Saint-Andiol (St-A) in the Bouches-du-Rhône region of France. Its resistance is characterized as dominant and carried by the Z sex chromosome (type I resistance). This colony remains susceptible to other isolates, such as CpGV-R5 [31].

### 2.2. Viruses

CpGV-M is the first recorded CpGV isolate, discovered in Mexico and described by Tanada in 1964 [32]. Its genome is 123.5 kbp [33]. The isolate CpGV-R5 has been described previously [34]. Its genome size is 123.1 kbp. Both viruses were produced on CpNPP larvae following the same protocol. In CpNPP, CpGV-M and CpGV-R5 LC_50_s are 13.10 (6.55–23.20), and 6.76 (2.6–13.37) OB·µL, respectively, while in R_GV_, CpGV-M and CpGV-R5 LD_50_s are 2.22 × 10^6^ (1.19 × 10^6^–5.67 × 10^6^), and 22.43 (13.73–34.36) OB·µL, respectively [35].

CrpeNPV is an SNPV isolated in 2018 in South Africa from *C. peltastica* (litchi moth). It has a genome of 115,728 bp (GenBank accession numbers: MH394321) [29]. The CrpeNPV isolate was provided by Rhodes University in South Africa. It was amplified on CpNPP larvae.

OB concentrations were estimated by counting on dark field light microscopy as previously described [31].

### 2.3. Infection Categories and Virus Processing

#### 2.3.1. Order of Infection

Analysis of the order of infection was performed on R_GV_ larvae. Three independent assays were carried out. CpGV-M and CpGV-R5 viruses were deposited on different dishes of Stonefly Heliothis medium (20 cm²) at a concentration of 10^4^ OB·cm^−2^. Neonate larvae were placed onto the surface of the 1st and then the 2nd virus treated medium (CpGV-M or CpGV-R5), for various durations (30, 60, 120 and 240 min). The larvae were then transferred to non-treated medium for 3 days. The larvae were then crushed individually and the virus resuspended into 200 µL of distilled water and analyzed by quantitative polymerase chain reaction (qPCR) coupled to high-resolution melting (qPCR/HRM) (see Section 2.4 below).

#### 2.3.2. Delay of Ingestion

R_GV_ neonate larvae were first allowed to feed on CpGV-R5 treated medium for 30 min (time necessary for ingestion of sufficient virus to result in double infection [36]). Larvae were then transferred to non-treated medium and left for various durations (Xi), then retransferred to the medium inoculated with CpGV-M for 30 min, and finally transferred back to non-treated medium for 3 days. Three independent experiments were carried out. The infection status of larvae was established by qPCR/HRM (see Section 2.4 below).

#### 2.3.3. Helper Effect Specificity

It has been demonstrated that CrpeNPV is able to infect various codling moth colonies [30]. The ability of CrpeNPV to infect CpNPP and R_GV_ was first verified (data not shown).

To check if double infections can occur using two virus species, neonate CpNPP larvae were exposed to medium (Stonefly Heliothis medium) inoculated with a virus mixture (50% CrpeNPV and 50% CpGV-M, or 50% CrpeNPV and 50% CpGV-R5), with final concentration of 10^4^ OB·cm^−2^. At 3 days, the larvae were collected and crushed into 200 µL. Each sample was split into two separate qPCR reactions and each amplicon was identified by HRM analysis.

Lastly, to check if CrpeNPV can substitute CpGV-R5 for helping CpGV-M replication in resistant larvae, R_GV_ neonate larvae were fed with a mixture of 50% CrpeNPV and 50% CpGV-M, and processed under similar conditions. Similarly, R_GV_ third instars were fed with a mixture of 50% CrpeNPV and 50% CpGV-M with 2.22 × 10^4^ OB·cm^−2^, collected at 4 days and processed under similar conditions.

### 2.4. Quantitative Polymerase Chain Reaction (qPCR) and High-Resolution Melting (HRM) Analysis

qPCR was carried out in the conditions previously described [37]. CpGV-M and CpGV-R5 isolates were identified by qPCR, targeting the *pe38* gene region using the primers CpGV-18734F (5′-GCCACCATTAGTGAATCATC-3′) and the reverse primer CpGV-18855R (5′-TAAGTCAGGACACCCAAACC-3′). The *pe38* gene is involved in type I resistance of *C. pomonella* [38]. CpGV-M and CpGV-R5 isolates produce a 121 bp and a 97 bp amplicons respectively.

CrpeNPV was identified using the primers polh_CrpeNPV_F (5′-CGAGCATGAAATCGAGGAAC-3′) and polh_CrpeNPV_R (5′-ACTTCGTGAGGCACATAGTC-3′) targeting the polyhedrin region (GenBank accession no.MH394321) [29]. CrpeNPV produces a 342 bp amplicon.

Each amplicon produced by the different viruses was submitted to HRM analysis. A melting curve step was added: 5 s at 95 °C then 10 s at 50 °C and increasing the temperature from 70 to 90 °C by steps of 0.2 °C, maintained for 10 s each. The software Biorad CFX maestro™ and Precision Melt Analysis™ were used for interpretation. To avoid possible interferences, amplifications of CpGV and CrpeNPV were carried out separately.

## 3. Results

### 3.1. Importance of the Order of Infection with CpGV-M and CpGV-R5

CpNPP larvae are susceptible to all viruses tested. R_GV_ larvae are resistant to the CpGV-M isolate and none were found infected with CpGV-M alone; they are susceptible to the CpGV-R5 isolate. In our experimental conditions, the proportion of larvae infected with CpGV-R5 varied from 50% to 80%, regardless of the order of ingestion.

**Figure 1:**
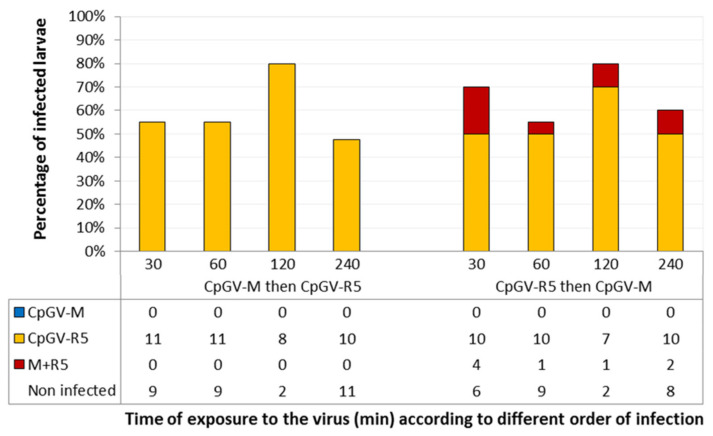
Infection status determined by quantitative polymerase chain reaction (qPCR) of R_GV_ larvae (*n* ≈ 150) inoculated sequentially with both viruses for the durations indicated. Three experiments were carried out. The cumulative numbers of larvae infected by CpGV-M or CpGV-R5 or both CpGV-M and CpGV-R5 (labelled M + R5) are shown in the table.

On R_GV_, when CpGV-M was ingested first, followed by CpGV-R5, no CpGV-M infection was observed. Conversely, if CpGV-R5 was ingested first, 5% to 20% of individuals were infected with both viruses (Figure 1).

### 3.2. Delay between the Ingestion of the Different Genotypes

The importance of a delay between the ingestion of the first isolate, CpGV-R5, and the second, CpGV-M was analyzed. For this purpose, increasing time-lapses between exposures to the two viruses were tested.

**Figure 2:**
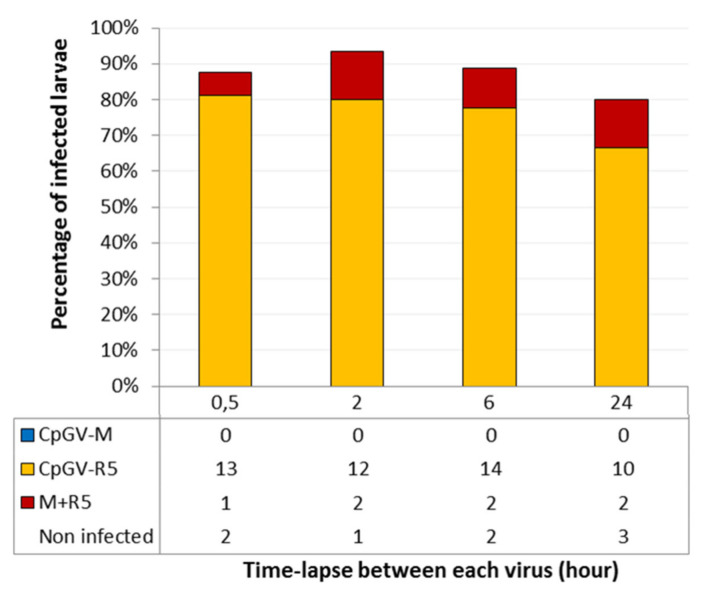
Infection status determined by qPCR of R_GV_ larvae (*n* ≈ 80) inoculated sequentially with CpGV-R5, then with CpGV-M, in function of the time-lapses between exposure to different genotypes (in hours). Three experiments were carried out. The cumulative number of larvae infected by CpGV-M, CpGV-R5 or both CpGV-M and CpGV-R5 (labelled M + R5) are shown in the table.

A contact time with the viruses of 30 min was chosen, as the levels of infection were satisfactory for the concentration of 10^4^ OB·cm^−2^. The percentage of larvae infected with only CpGV-R5 varied between 67% and 80%. The percentage of larvae infected with both viruses varied between 6% and 13%, regardless of the time-lapse between exposure to each of the two viruses (Figure 2). These results suggest that there is no dependence on the delay of ingestion of the second virus to obtain a double infection. Once the CpGV-R5 virus is ingested, the CpGV-M virus can replicate in larvae with type I resistance.

### 3.3. Double Infections Using CrpeNPV

*C. pomonella* CpNPP (CpGV-M permissive) larvae were allowed to feed on mixtures of CrpeNPV and CpGV-M. The replication of each virus was detected using qPCR followed by HRM. The mean Tm (melting temperature) of CpGV-M and CrpeNPV amplicons were 81.8 °C and 84.4 °C, respectively, and their size was verified by electrophoresis (data not shown). The two peaks are clearly distinct, allowing non ambiguous identification of each infection. Each larva was analyzed separately. Replication of GV and NPV was detected in more than 70% of the CpNPP infected larvae, for both CpGV genotypes (Table 1).

When R_GV_ larvae were fed on mixtures of CrpeNPV and CpGV-R5, two amplicons were obtained, corresponding to the specific signals of these two viruses (data not shown). As shown for CpNPP, it was found that R_GV_ larvae are permissive to multiple infections.

Finally, neonate R_GV_ larvae were inoculated with CpGV-M and CrpeNPV-SA isolates. No R_GV_ larva was found infected with CpGV-M in the presence of CrpeNPV, even when doubling the quantity of CpGV-M. Given the sensitivity of qPCR approaches, if CpGV-M replication had occurred, it certainly would have been detected. To check if the development stage was a factor influencing the multiple infection, a similar experiment was carried out with third instar R_GV_ larvae. No double infection was detected.

## 4. Discussion

Helper activities have been previously described in other baculovirus/host systems. Tanada (1959) described how the presence of a GV allowed replication of PsunNPV in the armyworm *Pseudaletia unipuncta*, while the NPV alone could not replicate [39]. Later it was demonstrated that NPV was blocked at the peritrophic membrane, and the GV helped passing through this barrier in a *trans* complementation manner [40,41,42]. Supplementing NPV with GV proteins resulted in NPV being able to infect orally. Infection was also possible when NPV was injected into the haemocoele of the larvae [43]. A different situation was described in *Spodoptera littoralis* NPV (SpliNPV). *pif-1* deficient genomes can infect orally only if the virions contain active PIF-1 protein, that is, a *cis* complementation. Co-infection allowed *pif-1* deficient genomes to be enveloped with PIF-1 containing membranes due to the presence in the same cell of complete genomes [44].

The point of blockage of CpGV-M replication on type I resistant codling moth larvae has been studied by Asser-Kaiser et al. (2011). Replication is blocked independently of the mode of entry, by ingestion or by injection. CpGV-M is able to enter into the cells, but its replication is stopped at an early stage prior to DNA replication. These authors concluded that the resistance is not restricted to the primary infection in the midgut, but all cells in the larvae appear to be restrictive for the replication of CpGV-M [27].

Previous work on multiple infections has shown that the CpGV-R5 isolate allows replication of the CpGV-M isolate in the non-permissive R_GV_ host [28]. Our present results confirm these findings, and demonstrate that the infection of the larva by CpGV-R5 should be prior to the infection by CpGV-M. As the blocking point is after the entry in the cells, is a prior infection with CpGV-R5 necessary in order for a cell to be permissive to CpGV-M? At later stages of the infection, most cells of the larvae have been infected. However, in primary infections, the number of midgut cells is extremely large compared to the number of virions available. Consequently, infection of a midgut cell is a rare event, even with high doses of NPV, where each OB liberates many virions [45]. It can be expected that with GV, containing mainly only one virion, this event would be even rarer. If midgut infection in the codling moth follows similar rules, the probability of a cell being infected by two viruses is remote and cannot account for the number of double infections detected.

If a midgut cell needs to be infected by CpGV-R5 prior to CpGV-M co-infection to allow replication of the latter, the total number of midgut cells infected should be very important to result in 10% to 20% double infections (Figure 1), even if midgut cell renewal in codling moth larvae was low. Our results do not confirm this proposition. We have used infection doses (10^4^ OB·µL) that do not reach 100% mortality to avoid such a massive midgut infection.

In the systems that have been studied, midgut infection appears to be transient, that is, infected cells are lost by sloughing and no infection from cell to cell was observed in the midgut [45,46,47]. The life span of intestinal cells has been evaluated to be less than 8 h in other insects. We consider that a 24 h experiment should allow enough sloughing in CM. Increasing the delay between ingestion of the two viruses should reduce the probability of double infections. If midgut cells could be infected by direct transmission from neighboring infected cells, the proportion of double infections would increase when increasing the delay between exposure to the two viruses, but no such increase was observed. Accordingly, either there is no need for each cell to be infected by CpGV-R5 for supporting CpGV-M replication, or previous infection of a midgut cell by CpGV-R5 increases the probability of infection by CpGV-M (that is, the infections with the two viruses become non-independent events).

In cell culture, it has already been shown for SfMNPV that the probability of a successful infection with a second virus is related to the time-lapse between the first and second infection, referred to as the window of superinfection [48]. The time-lapse is the consequence of the reorganisation of the actin filaments in the cell. Once the virus enters the cell and the nucleocapsid is released into the cytoplasm, the nucleocapsid migrates to the nucleus using the actin filaments [48,49]. These filaments are later used for the migration of newly formed nucleocapsids in the nucleus to the cytoplasm to generate BV. Between these two steps, the orientation of the filaments changes, thus preventing the migration of new infecting viruses (new nucleocapsids from the membrane to the nucleus). Superinfection of the cells is only possible during a time-lapse constrained by the reorganisation of the actin filaments. In the *Cydia pomonella*/CpGV model and with the tools currently available, it is only possible to determine the duration of the ingestion delay allowing a double infection and not the existence of a superinfection time-lapse, which is only accessible at the cell scale. For these hypotheses to be tested, it will be necessary to follow the infection of each individual virion in the midgut. Such an observation will be possible once the ANCHOR^TM^ system we have previously adapted to AcMNPV [50] is transposed to CpGV. This research is ongoing.

It has been demonstrated that CrpeNPV is able to replicate in four different groups of *C. pomonella* larvae, carrying diverse resistance genes to CpGV, CpS, CpRR1, CpR5M, and CpRGO colonies [30]. We have verified that in our two colonies, CpNPP and R_GV_, this virus also replicates efficiently. We have used this second virus species (CrpeNPV) to test if the helper effect of CpGV-R5 could be substituted by another virus that replicates in the larvae. We first determined that double infections can occur using CrpeNPV and CpGV-M in fully permissive CpNPP larvae and CrpeNPV and CpGV-R5 in R_GV_ larvae. Subsequently, the occurrence of double infection was analyzed when a mixture of CrpeNPV and CpGV-M was given to R_GV_ larvae. No double infections were found, suggesting that CrpeNPV cannot help CpGV-M to replicate. Probably other CpGV genotypes could act as helpers, but this has not yet been tested.

We have previously proposed [28] that a diffusible factor secreted by CpGV-R5 infected cells could make the R_GV_ cells susceptible to CpGV-M. This theory would explain the results observed in the present experiments. If such a factor exists, the absence of helper activity of CrpeNPV would suggest that it is specific.

## 5. Concluding Remarks

Codling moth resistance to CpGV genotypes is a major threat for apple and pear producers. The presence of multiple CpGV genotypes in the virus inoculum results in it not behaving as single genotypes do. The interactions between CpGV genotypes appear to be specific and cannot be substituted by the presence of more distant viruses infecting the same host. The analysis of these interactions opens the way to a more detailed understanding of the early steps of the infection process.

## Figures and Tables

**Table 1:** Replication of virus inocula on the two insect host colonies. Number of larvae showing amplification of the specific PCR fragment after ingestion of the corresponding virus or virus mixture.

		Virus Replication					
Host	Virus Inoculum	CpGV-M	CpGV-R5	CrpeNPV	CpGV-M + CrpeNPV	CpGV-R5 + CrpeNPV	Total Number of Larvae
CpNPP	CpGV-M	11	-	-	-	-	12
	CpGV-R5	-	8	-	-	-	12
	CpGV-M + CrpeNPV	1	-	2	11	-	15
	CpGV-R5 + CrpeNPV	-	2	2	-	10	15
R_GV_	CpGV-M	0	-	-	-	-	13
	CpGV-R5	-	20	-	-	-	25
	CpGV-M + CrpeNPV	0	-	19	0	-	30
	CpGV-R5 + CrpeNPV	-	7	1	-	7	22
